# Apolipoprotein A1 and serum amyloid A in dogs with sepsis and septic shock

**DOI:** 10.3389/fvets.2023.1098322

**Published:** 2023-03-02

**Authors:** Cecilia Bulgarelli, Elena Ciuffoli, Roberta Troia, Robert Goggs, Francesco Dondi, Massimo Giunti

**Affiliations:** ^1^Department of Veterinary Medical Sciences, Alma Mater Studiorum—University of Bologna, Bologna, Italy; ^2^Department of Clinical Sciences, College of Veterinary Medicine, Cornell University, Ithaca, NY, United States

**Keywords:** high-density lipoproteins, acute phase protein, canine, organ dysfunction, lactate

## Abstract

**Introduction:**

Apolipoprotein-A1 (Apo-A1) acts as a negative acute phase protein (APP) during inflammatory states, and has a potential prognostic value in people and dogs with sepsis. The aim of this retrospective study was to investigate the association of serum Apo-A1 concentration with disease severity, multiorgan dysfunction syndrome (MODS) and outcome in a population of dogs with sepsis, and to assess its correlation with major canine APPs.

**Methods:**

Ninety-nine dogs with uncomplicated sepsis (*n* = 78) or septic shock (*n* = 21) were included. The serum concentration of Apo-A1, C-reactive protein (CRP) and serum amyloid A (SAA) were recorded, alongside the canine acute patient physiologic and laboratory evaluation fast (APPLE_fast_) score and the presence of MODS.

**Results:**

Dogs with septic shock had significantly lower serum Apo-A1 concentrations (106.3 ± 22.7 mg/dl; reference interval: 123.0–142.3 mg/dl), higher APPLE_fast_ score (30, 13–38) and greater frequency of MODS (67%) compared to those with uncomplicated sepsis (117.9 ± 19.3 mg/dl; 25, 6–33 and 8%, respectively) (*P* = 0.0201; *P* = 0.0005; P < 0.0001, respectively). Similarly, dogs with MODS had significantly lower serum Apo-A1 concentrations (104.1 ± 4.6 mg/dl) and higher APPLE_fast_ score values (31, 13–38) compared to those without MODS (118.32 ± 2.1 mg/dl and 26, 6–33, respectively) (*P* = 0.0050 and *P* = 0.0038, respectively). Conversely, neither CRP nor SAA were different between these groups. No difference in serum APPs concentrations was detected between survivors and non-survivors. Significant negative correlations were detected between serum Apo-A1 and SAA (*P* = 0.0056, *r* = −0.277), and between serum Apo-A1 and the APPLE_fast_ score (*P* = 0.0027, *r* = −0.3). In this population, higher values of the APPLE_fast_ score and the presence of MODS were independently associated with a higher risk of death.

**Discussion:**

Our study shows that Apo-A1 is a useful biomarker of sepsis severity in dogs, since it is decreased in those with septic shock and MODS. Further prospective investigations are deemed to evaluate the applicability of Apo-A1 to predict sepsis course and response to treatment in septic dogs.

## Introduction

Apolipoprotein A1 (Apo-A1) is a major structural protein of plasma high-density lipoproteins (HDL) that is fundamental to HDL formation. The primary function of HDL is to mediate cholesterol homeostasis by facilitating its transport to the liver, but HDL is pleiotropic and modulates the inflammatory response during sepsis (1) by binding circulating lipopolysaccharides (LPS) and directly inactivating bacterial endotoxins (2). During systemic inflammatory states and sepsis in humans, decreased plasma Apo-A1 and HDL concentrations and reduced HDL function occur (3). Low Apo-A1 concentrations at hospital admission are associated with increased mortality, prolonged intensive care stay, and hospital-acquired infections in humans (4, 5).

Few data are available in dogs, but Apo-A1 is a potential biomarker of canine systemic inflammatory response syndrome (SIRS) and sepsis that behaves as a negative acute phase protein (APP) (6, 7). The exact mechanism by which infection and inflammation decrease Apo-A1 levels is unclear and likely multifactorial. Increased hepatic synthesis of serum amyloid A (SAA) plays a major role through binding to HDL, displacing Apo-A1 and accelerating HDL clearance during the acute phase response (8). Serum amyloid A is a major APP in humans and dogs and its serum concentration increases in septic patients, correlating with disease severity and outcome comparable to other markers including C-reactive protein (CRP) (9). In dogs, SAA concentration is significantly increased in SIRS and sepsis (10–14) but is not prognostic. Conversely, preliminary data suggested that Apo-A1 has potential prognostic value in dogs with sepsis and might act as a marker of disease severity in a specific setting of critical abdominal illness, like septic peritonitis (15). These findings need to be confirmed in a wider population of septic dogs classified according to disease severity.

Thus, the aim of our study was to evaluate the prognostic value of serum Apo-A1 concentration in dogs with sepsis by assessing its association with disease severity and outcome. We hypothesized that Apo-A1 behaves as a negative APP and inversely correlates with major canine APPs (SAA and CRP). Furthermore, we hypothesized that lower serum concentrations of Apo-A1 are associated with greater sepsis severity, documented by occurrence of either septic shock or multiple organ dysfunction and with non-survival.

## Materials and methods

### Animals

Dogs examined at the University of Bologna veterinary teaching hospital between 05/2018 and 10/2019 meeting inclusion criteria were considered eligible for study. The study protocol was approved by the local Institutional Animal Care and Use Committee (protocol number ID 846). Dogs were considered eligible if they were diagnosed with sepsis based on the presence of infection confirmed by cytology, microbiology, histopathology, or real-time polymerase chain reaction, and satisfied at least 2/4 SIRS criteria, as previously reported (15). Dogs were included in the study if they were hospitalized in the intensive care unit (ICU) for at least 12 h and an aliquot of serum collected upon hospital admission and stored frozen (−80°C) was available. Enrolled dogs were subsequently classified as uncomplicated sepsis or septic shock, wherein septic shock was defined by either persistent hyperlactatemia (lactate >2 mmol/L for >12 h despite fluid resuscitation or clinical euvolemia) or hypotension (systolic blood pressure, SAP <90 mmHg) requiring vasopressor support despite adequate fluid resuscitation (16, 17). Dogs with septic shock were sub-classified by septic shock phenotype as cryptic shock (persistent hyperlactatemia without fluid-refractory hypotension), vasoplegic shock (fluid-refractory hypotension with normal blood lactate) or dysoxic shock (persistent hyperlactatemia and fluid-refractory hypotension) (16, 18). Dogs were excluded if they had an intercurrent disease likely to influence Apo-A1 concentration such as chronic liver disease, hyperlipidemia, or if they were receiving total parenteral nutrition.

### Data collection

The following variables were recorded at the time of hospital admission: signalment, history, bodyweight, previous and ongoing treatments, rectal temperature, heart rate, respiratory rate, mentation status, and non-invasive systolic and mean arterial pressure (SunTech^®^ Vet20^TM^ Veterinary Blood Pressure Monitor, SunTech Medical, Inc., USA; Minidop ES-100 VX, Hadeco, Kawasaki, Japan). Attending clinicians were responsible for the clinical management of dogs included in the study. Blood was collected by venipuncture into evacuated tubes according to standard operating procedures and analyses performed at the institutional clinical pathology laboratory. Blood gas, electrolytes, and blood lactate measurements were performed using a point-of-care analyzer (ABL800 FLEX, Radiometer Medical ApS, Denmark). Complete blood count and serum chemistry analyses were performed using automated analyzers (ADVIA 2120, Siemens Healthcare Diagnostics, Tarrytown, NY; Olympus AU 480, Olympus/Beckman Coulter, Brea, CA). Coagulation assays were performed on citrated plasma using a benchtop automated analyzer (SEAC Clot 2S and BTF II, Siemens, Marburg, Germany). Patient data were used to calculate the rapid canine Acute Patient Physiologic and Laboratory Evaluation (APPLE_fast_) score to assess disease severity (19).

### Apo-A1, CRP, and SAA measurement

The Apo-A1 immunoassay was calibrated (Apo-A1 and B calibrator; Olympus/Beckman Coulter, O'Callaghan's Mills, Ireland) using standards and quality control materials provided by the manufacturer (Control serum L1 and L2; Olympus/Beckam Coulter, O'Callaghan's Mills, Ireland). An internal validation was performed (intra- and inter-assay coefficients of variation < 5%; spiking recovery 80–120%) (15). CRP was measured by using an immunoturbidimetric assay (CRP OSR6147, Olympus/Beckman Coulter, O'Callaghan's Mills, Ireland) that had been previously validated by our group for canine serum samples (CV of 5.8%; detection limit of 0.01 mg/dl) (20). Quantitation of SAA concentrations was performed at the Clinical Pathology Laboratory of Cornell University (CU) using a validated latex turbidimetric immunoassay (Vet-SAA Eiken, Eiken Chemical CO, LTD, Japan). Intra- and inter-assay CVs ranged from 1.9% to 4.1% and from 9.7% to 12.5%, respectively, in measurements of high and intermediate concentrations of canine SAA. Acceptable linearity was observed within a clinically relevant working range (0–1,000 mg/L) (21). For this purpose, serum samples were shipped to CU on dry ice by overnight courier. All samples were frozen on arrival and were stored at −80°C prior to batch analysis (12, 22, 23).

### Organ dysfunction and outcome

The presence or absence of multiorgan dysfunction syndrome (MODS), defined as the presence of at least two dysfunctional organs simultaneously, was determined as previously described (24). Dogs were classified as survivors if they were discharged from the hospital alive or non-survivors if they died despite medical treatment or were humanely euthanized due to moribund conditions or end-stage disease. Dogs euthanized for financial reasons were excluded from the study.

### Statistical analysis

Data distribution was assessed graphically and using the D'Agostino Pearson test. Data were reported as median and range (minimum-maximum), or mean ± standard deviation (SD), based on their distribution. The Mann-Whitney U-test, Student's t-test and the Kruskal-Wallis test with *post-hoc* comparison according to Conover were used to compare continuous variables among groups (uncomplicated sepsis vs. septic shock; dogs with vs. without MODS; survivors vs. non-survivors; underlying diseases and different subgroups of septic shock). Categorical variables were compared among groups using Fisher's exact test or the Chi squared test. The Spearman correlation coefficient was used to identify significant correlations between variables. Receiver operating characteristic (ROC) curve analysis was used to find optimal cut-off values for variables (serum Apo-A1, CRP, SAA, and APPLE_fast_ score) predicting the presence of septic shock and development of MODS, and to calculate the area under the ROC curve (AUC). Associations between variables of interest (serum albumin, Apo-A1, APPLE_fast_ score, serum creatinine, CRP, SAA, blood ionized calcium, blood lactate, and presence of MODS) and survival for the overall population of septic dogs were examined by univariate regression analysis, and variables associated with the outcome (*P* ≤ 0.1) were included in a multivariable regression model (stepwise selection). Results are presented as odds ratio (OR) with 95% confidence intervals (CI). For all hypothesis tests, values of *P* < 0.05 were considered significant. Statistical analyses were performed using commercial software (MedCalc Statistical Software version 19.1.3 Ostend, Belgium 2019).

## Results

The study included 99 dogs; 78 (79%) had uncomplicated sepsis and 21 (21%) had septic shock. Among dogs with uncomplicated sepsis, there were 13/78 (17%) spayed females, 35/78 (45%) intact females, 7/78 (9%) neutered males and 23/78 (29%) intact males. Thirty-five breeds were represented, with the most common being mixed breed (*n* = 21), Labrador Retriever (*n* = 5), Jack Russell Terrier (*n* = 5), German Shepherd (*n* = 4), Golden Retriever (*n* = 3) and Cocker Spaniel (*n* = 3). Median age was 7 years (0.1–16) and the median bodyweight was 18 kg (0.4–59.8). Underlying causes for uncomplicated sepsis included reproductive tract infection (pyometra, *n* = 22; metritis, *n* = 4), respiratory tract infection (pneumonia, *n* = 8; pulmonary abscess, *n* = 3; pyothorax, *n* = 1), parvoviral enteritis (*n* = 9), skin and subcutaneous tissue infections (bite wounds, *n* = 7; necrotizing fasciitis, *n* = 2), urinary tract infections (pyelonephritis, *n* = 5; prostatic abscess, *n* = 3), intra-abdominal infections (septic peritonitis, *n* = 4; pancreatic abscess, *n* = 1; penetrating bite wound, *n* = 1), miscellaneous diseases (leptospirosis, *n* = 2; bacteremia associated with hemorrhagic gastroenteritis, *n* = 2; discospondylitis, *n* = 1; endocarditis, *n* = 1; rectal laceration, *n* = 1; purulent lymphadenitis, *n* = 1). Among dogs affected by septic shock, there were 4/21 (19%) spayed females, 7/21 (33%) intact females, 2/21 (10%) neutered males and 8/21 (38%) intact males. Median age was 10.5 years (0.4–14) and the median bodyweight was 11.8 kg (4.3–37.8). Underlying causes for septic shock included septic peritonitis (*n* = 6), bite wound (*n* = 5), urinary tract infection (*n* = 4), pyometra (*n* = 2), pneumonia (*n* = 2), parvoviral enteritis (*n* = 1), bacteremia associated with hemorrhagic gastroenteritis (*n* = 1). Phenotypic classification suggested 7/21 (33%) dogs had cryptic shock, 12/21 (57%) dysoxic shock and 2/21 (10%) vasoplegic shock. All dogs with dysoxic and vasoplegic septic shock (14/21, 67%) received norepinephrine, and 6/14 (43%) also received dobutamine.

**Figure 1:**
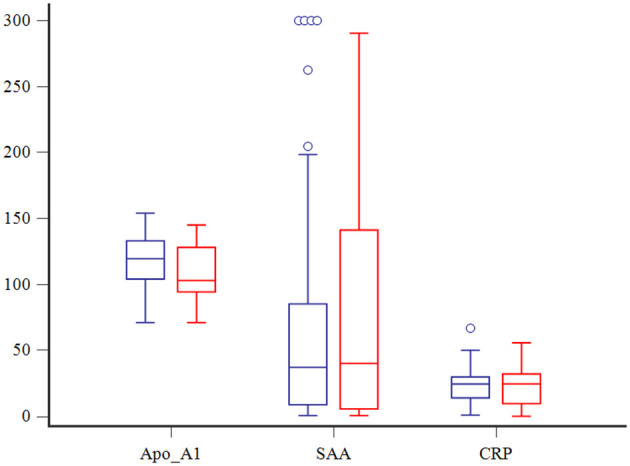
Box and whisker plots comparing Apo-A1 (mg/dl), SAA (μg/dl) and CRP (mg/dl) among dogs with (in red) and without (in blue) septic shock. The central lines represent the median, the boundaries of the boxes represent the interquartile range and the whiskers represent the minimum and maximum values. Blue circles indicate outliers.

Clinical and clinicopathologic findings in dogs with sepsis and septic shock are summarized in Table 1. Mean Apo-A1 serum concentrations were significantly lower in dogs with septic shock compared to those with uncomplicated sepsis, while mean CRP and median SAA serum concentrations were not significantly different between groups (Figure 1). No significant differences in the investigated APPs according to the underlying septic disease or the phenotype of septic shock were observed (data not shown). In the overall population of septic dogs, serum SAA and CRP concentrations were positively correlated (r_s_ = 0.71, *P* < 0.0001), while serum Apo-A1 and SAA concentrations were negatively correlated (r_s_ = −0.28, *P* = 0.0056). No significant correlation was found between serum Apo-A1 and CRP concentrations. Moreover, serum Apo-A1 and albumin were positively correlated (r_s_ = 0.58, *P* < 0.0001).

**Table 1:** Descriptive statistics of selected clinical and clinicopathological variables in dogs with sepsis and dogs with septic shock.

**Variable**	**RI**	**Sepsis (*n* = 78)**	**Septic shock (*n* = 21)**	***P*** **value**
**Clinical data**				
Body temperature (°C)	38–39	39.2 (35.5–41.3)	38.5 (33.0–40.9)	0.0224
Heart rate (bpm)	60–120	129 ± 26	157 ± 29	< 0.0001
Respiratory rate (rpm)	10–40	38 (20–160)	38 (16–68)	NS
SBP (mmHg)	120–170	141 ± 23	83 ± 28	< 0.0001
APPLE_fast_ score	NA	25 (6–33)	30 (13–38)	0.0005
Days of hospital stay	NA	3 (0–27)	5 (0–14)	NS
Lactate (mmol/L)	0.5–2.0	2.4 (0.4–4.8)	3.5 (1.6–9.9)	0.0003
**Hematology**				
HCT (%)	37.0–55.0	38.7 ± 8.8	42.0 ± 7.6	NS
WBCs (× 10^9^/L)	6.0–17.0	17.98 (0.90–79.19)	8.15 (0.95–79.75)	0.0008
Platelets (× 10^9^/L)	160–500	254 (23–729)	239 (29–823)	NS
**Chemistry**				
Glucose (mg/dl)	65–115	103 (52–486)	115 (20–665)	NS
Creatinine (mg/dl)	0.75–1.40	0.88 (0.28–14.80)	1.33 (0.23–9.43)	NS
Total bilirubin (mg/dl)	0.07–0.33	0.22 (0.04–107.00)	0.23 (0.01–2.01)	NS
Albumin (mg/dl)	2.75–3.85	2.46 ± 0.49	2.13 ± 0.56	0.0106
Total proteins (mg/dl)	5.60–7.30	6.14 ± 1.21	5.45 ± 1.13	0.0228
**Coagulation**				
PT (s)	5.0–7.5	7.0 (5.4–10.0)	7.2 (5.8–14.0)	NS
aPTT (s)	8.0–16.5	13.0 (7.5–36.6)	13.6 (9.6–21.5)	NS
**Acute phase proteins**				
Apo-A1 (mg/dl)	123.0–142.3	117.9 ± 19.3	106.3 ± 22.7	0.0201
SAA (μg/ml)	5–200	370 (5–3,000)	400 (5–2,905)	NS
CRP (mg/dl)	0.0–0.85	22.3 ± 13.2	23.6 ± 15.5	NS

Data are reported as mean ± standard deviation or median and range (minimum–maximum value), based on their distribution. Apo-A1, Apolipoprotein A1; APPLE, acute patient physiologic and laboratory evaluation; aPTT, activated partial thromboplastin time; CRP, C-Reactive Protein; HCT, hematocrit value; PT, prothrombin time; RI, reference interval; SAA, Serum Amyloid A; SBP, systolic blood pressure; WBCs, white blood cells; NA, not applicable/not available; NS, not significative.

**Figure 2:**
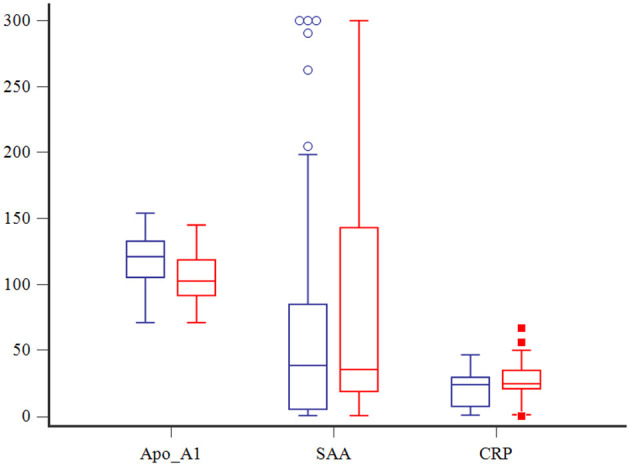
Box and whisker plots comparing Apo-A1 (mg/dl), CRP (mg/dl) and SAA (μg/dl) among dogs with (in red) and without (in blue) MODS. The central lines represent the median, the boundaries of the boxes represent the interquartile range and the whiskers represent the minimum and maximum values. Blue circles and red squares indicate outliers.

The APPLE_fast_ illness severity scores were significantly higher in dogs with septic shock compared to those with uncomplicated sepsis, and APPLE_fast_ scores were negatively correlated with Apo-A1 serum concentrations (r_s_ = −0.3, *P* = 0.0027). No significant correlations between APPLE_fast_ scores or CRP or SAA concentrations were observed. The frequency of MODS was significantly lower in dogs with uncomplicated sepsis (6/78, 8%) compared to those with septic shock (14/21, 67%, *P* < 0.0001). Dogs with MODS had significantly lower Apo-A1 concentrations compared to those without MODS (104.1 ± 4.6 vs. 118.32 ± 2.1, *P* = 0.0050), and significantly higher APPLE_fast_ scores (31, 13–38 vs. 26, 6–33, *P* = 0.0038). Serum CRP and SAA concentrations were not discriminating for MODS (Figure 2).

According to the ROC curve analysis, a serum Apo-A1 ≤ 104 mg/dl had a 57% sensitivity and a 74% specificity (AUC = 0.65) to correctly predict septic shock (*P* = 0.04), and a 65% sensitivity and a 76% specificity (AUC = 0.70) to correctly predict MODS (*P* = 0.004), respectively. Furthermore, an APPLE_fast_ score >26 had an 81% sensitivity and a 64% specificity (AUC = 0.75) to correctly predict septic shock (*P* = 0.0003), while an APPLE_fast_ score >30 had a 55% sensitivity and an 88% specificity (AUC = 0.71) to correctly predict MODS (*P* = 0.009).

**Figure 3:**
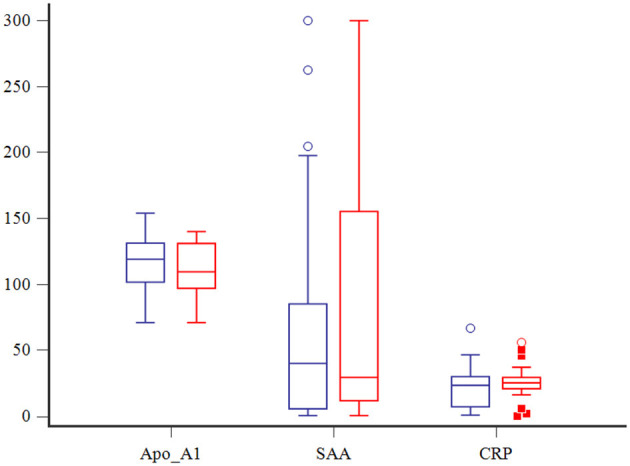
Box and whisker plots comparing Apo-A1 (mg/dl), CRP (mg/dl) and SAA (μg/dl) among survivors (in blue) and non-survivors (in red). The central lines represent the median, the boundaries of the boxes represent the interquartile range and the whiskers represent the minimum and maximum values. Circles and squares indicate outliers.

The overall case fatality rate was 26% (26/99) and was higher in dogs with septic shock 52% (11/21) than in dogs with uncomplicated sepsis 19% (15/78, *P* = 0.0043). Among non-survivors, 16/26 (62%) dogs were humanely euthanized. No differences in serum concentrations of Apo-A1, CRP and SAA between survivors and non-survivors were observed (Figure 3). Univariate analyses suggested that presence of MODS, increased APPLE_fast_ score, serum creatinine, and blood lactate, and decreased blood ionized calcium were associated with non-survival. Of these, multivariable logistic regression analysis indicated that increased APPLE_fast_ score and the presence of MODS were independently associated with non-survival (Table 2).

**Table 2:** Logistic regression analysis of variables associated with outcome (survivors/non-survivors) in 99 dogs with sepsis.

**Variable**	**RC**	**SE**	**Odds ratio (95% CI)**	***P*** **value**
Serum creatinine (mg/dl)	0.84	0.28	2.31 (1.33–4.03)	**< 0.0001**
Ionized calcium (mmol/L)	−7.29	2.35	0.0007 (0.00–0.07)	**0.0019**
Blood lactate (mmol/L)	0.31	0.14	1.36 (1.03–1.80)	**0.030**
APPLE_fast_ score	0.14	0.05	1.16 (1.05–1.28)	**0.001**
Presence of MODS	2.24	0.56	9.43 (3.16–28.17)	**< 0.0001**

CI, confidence interval; RC, regression coefficient; SE, standard error; APPLE_fast_ score, acute patient physiologic and laboratory evaluation score; MODS, multiorgan dysfunction syndrome.

## Discussion

Critically ill patients with SIRS and/or sepsis present with alterations in lipid metabolism. The reduction in lipoprotein levels, including Apo-A1, is proportional to the degree of inflammation, and correlated upon ICU admission with increased severity, ICU time, hospital infection rate, and mortality in humans (25).

The results of the present study confirm that Apo-A1 behaves as a negative acute phase protein in dogs with sepsis, as previously reported (15). The latter statement is warranted by the reported moderate positive correlation between serum Apo-A1 and albumin. A weak, but significant inverse relationship between serum Apo-A1 and SAA concentrations was observed that may be due to replacement of Apo-A1 by SAA in the formation of acute-phase HDL (25). In humans with sepsis, strong associations between Apo-A1 and SAA have not been consistently reported (26), and controversial results were reported in a population of dogs with acute *Babesia canis* infection (27). No significant correlation was observed between serum CRP and Apo-A1 concentrations in our study, a result that was unexpected based on a prior study of dogs with leishmaniosis where Apo-A1 and CRP were inversely correlated (6). This may be due to distinct underlying pathophysiology and differences in the timing of blood sample acquisition relative to disease course and severity. Data on the kinetics of Apo-A1 concentrations in dogs with sepsis are lacking, hampering comparisons between studies. Further investigation of the time-course of Apo-A1 concentrations in dogs with sepsis are warranted.

The prognostic value of the APPLE_fast_ score for dogs with sepsis and SIRS is well-recognized (7, 28). Similarly, development of MODS is a frequent complication of sepsis in humans (29) and dogs (24) and is also strongly associated with mortality (24, 30). In our study, serum Apo-A1 and APPLE_fast_ score were able to discriminate sepsis severity as assessed by the presence of septic shock and development of MODS. Conversely, neither serum CRP or SAA had a significant predictive performance or correlated with APPLE_fast_ scores. This suggests that measurement of Apo-A1 in addition to, or in place of the major APPs may be of value in dogs with sepsis. However, differently from APPLE_fast_ score, serum Apo-A1 was not able to predict survival in this population of septic dogs. Additional studies in large heterogenous populations and in specific homogenous patient subgroups will be needed to determine if Apo-A1 is a valuable clinical biomarker.

Our study has some limitations. Persistent hyperlactatemia was defined as blood lactate >2 mmol/L based on the available veterinary literature (31, 32), but sampling technique and patient restraint might have artifactually increased blood lactate values, leading to misclassification. However, dogs with cryptic shock fulfilled more SIRS criteria, had higher APPLE_fast_ scores, and higher rates of MODS and mortality compared to dogs with uncomplicated sepsis suggesting they were more severely affected. The retrospective nature of the data collection and lack of standardized patient management, including drawing blood cultures, may have led to missing or incomplete data and inclusion of a heterogeneous study patient population. These factors, combined with the relatively small sample size likely limited statistical power. The heterogeneity of the underlying causes of sepsis may have influenced our results and make direct comparisons with previous studies more difficult (6, 15). As with many similar studies, biomarkers including Apo-A1 were only measured at the time of hospital admission, and were not monitored serially over time or in relation to treatment (6, 33). Further studies will be necessary before Apo-A1 can be considered for use guiding clinical decision-making in dogs with sepsis. As with many veterinary studies, euthanasia likely introduced a bias in the evaluation of survival despite excluding patients euthanized that were not considered to have end-stage disease or be moribund.

In summary, in dogs with sepsis Apo-A1 behaves as a negative acute phase protein and may be a valuable marker of illness severity. In the present study population, Apo-A1 was superior to CRP and SAA for the identification of dogs with septic shock and MODS, and might be useful for screening dogs deemed high-risk.

## Data availability statement

The raw data supporting the conclusions of this article will be made available by the authors, without undue reservation.

## Ethics statement

The animal study was reviewed and approved by the Animal Welfare Committee (COBA) of the Alma Mater Studiorum-University of Bologna (Bologna ID 846). Written informed consent was obtained from the owners for the participation of their animals in this study.

## Author contributions

RT and MG designed the study, analyzed data, co-wrote, and edited the manuscript. CB and RG analyzed data, co-wrote, and edited the manuscript. FD and EC edited the manuscript. All authors contributed to read and approved the final manuscript.

## References

[1] Tanaka S Couret D Tran-Dinh A Duranteau J Montravers P Schwendeman A. High-density lipoproteins during sepsis: from bench to bedside. Critical Care. (2020) 24:1–1. 10.1186/s13054-020-02860-3 32264946 PMC7140566

[2] Pirillo A Catapano AL Norata GD. HDL in infectious diseases and sepsis. In: von Eckardstein A Kardassis D editors. High Density Lipoproteins Handbook of Experimental Pharmacology. Cham: Springer (2015). p. 483–508. 10.1007/978-3-319-09665-0_15 25522999

[3] Catapano AL Pirillo A Bonacina F Norata GD. HDL in innate and adaptive immunity. Cardiovasc Res. (2014) 103:372–83. 10.1093/cvr/cvu150 24935428

[4] Chien J-Y Jerng J-S Yu C-J Yang P-C. Low serum level of high-density lipoprotein cholesterol is a poor prognostic factor for severe sepsis. Crit Care Med. (2005) 33:1688–93. 10.1097/01.CCM.0000171183.79525.6B 16096442

[5] Bermudes ACG de Carvalho WB Zamberlan P Muramoto G Maranhão RC Delgado AF. Changes in lipid metabolism in pediatric patients with severe sepsis and septic shock. Nutrition. (2018) 47:104–9. 10.1016/j.nut.2017.09.015 29429528

[6] Escribano D Tvarijonaviciute A Kocaturk M Cerón JJ Pardo-Marín L Torrecillas A. Serum apolipoprotein-A1 as a possible biomarker for monitoring treatment of canine leishmaniosis. Comp Immunol Microbiol Infect Dis. (2016) 49:82–7. 10.1016/j.cimid.2016.10.002 27865270

[7] Giunti M Troia R Bergamini PF Dondi F. Prospective evaluation of the acute patient physiologic and laboratory evaluation score and an extended clinicopathological profile in dogs with systemic inflammatory esponse syndrome. J Vet Emerg Crit Care. (2015) 25:226–33. 10.1111/vec.12257 25427754

[8] Filippas-Ntekouan S Liberopoulos E Elisaf M. Lipid testing in infectious diseases: possible role in diagnosis and prognosis. Infection. (2017) 45:575–88. 10.1007/s15010-017-1022-3 28484991

[9] Yu M-H Chen M-H Han F Li Q Sun R-H Tu Y-X. Prognostic value of the biomarkers serum amyloid A and nitric oxide in patients with sepsis. Int Immunopharmacol. (2018) 62:287–92. 10.1016/j.intimp.2018.07.024 30048858

[10] Viitanen SJ Lappalainen AK Christensen MB Sankari S Rajamäki MM. The utility of acute-phase proteins in the assessment of treatment response in dogs with bacterial pneumonia. J Vet Intern Med. (2017) 31:124–33. 10.1111/jvim.14631 28032360 PMC5259651

[11] Ceron JJ Eckersall PD Martinez-Subiela S. Acute phase proteins in dogs and cats: current knowledge and future perspectives. Vet Clin Pathol. (2005) 34:85–99. 10.1111/j.1939-165X.2005.tb00019.x 15902658

[12] Christensen MB Langhorn R Goddard A Andreasen EB Moldal E Tvarijonaviciute A. Comparison of serum amyloid A and C-reactive protein as diagnostic markers of systemic inflammation in dogs. Can Vet J. (2014) 55:161. 24489396 PMC3894877

[13] Jitpean S Pettersson A Höglund OV Holst BS Olsson U Hagman R. Increased concentrations of serum amyloid A in dogs with sepsis caused by pyometra. BMC Vet Res. (2014) 10:1–9. 10.1186/s12917-014-0273-9 25430894 PMC4247870

[14] Jitpean S Holst BS Höglund OV Pettersson A Olsson U Strage E. Serum insulin-like growth factor-I, iron, C-reactive protein, and serum amyloid A for prediction of outcome in dogs with pyometra. Theriogenology. (2014) 82:43–8. 10.1016/j.theriogenology.2014.02.014 24661434

[15] Giunti M Grossi G Troía R Fracassi F Dondi F. Evaluation of serum apolipoprotein A1 in canine sepsis. Fron Vet Sci. (2020) 7:263. 10.3389/fvets.2020.00263 32478112 PMC7238865

[16] Ranzani OT Monteiro MB Ferreira EM Santos SR Machado FR Noritomi DT. Reclassifying the spectrum of septic patients using lactate: severe sepsis, cryptic shock, vasoplegic shock and dysoxic shock. Rev Bras Ter Insensiva. (2013) 24:270–8. 10.5935/0103-507X.20130047 24553507 PMC4031869

[17] Singer M Deutschman CS Seymour CW Shankar-Hari M Annane D Bauer M. The third international consensus definitions for sepsis and septic shock (Sepsis-3). JAMA. (2016) 315:801–10. 10.1001/jama.2016.0287 26903338 PMC4968574

[18] Troia R Buzzurra F Ciuffoli E Mascalzoni G Foglia A Magagnoli I. Classification of septic shock phenotypes based on the presence of hypotension and hyperlactatemia in cats. Fron Vet Sci. (2021) 8:6692528. 10.3389/fvets.2021.692528 34595228 PMC8476852

[19] Hayes G Mathews K Doig G Kruth S Boston S Nykamp S. The acute patient physiologic and laboratory evaluation (APPLE) score: a severity of illness stratification system for hospitalized dogs. J Vet Intern Med. (2010) 24:1034–47. 10.1111/j.1939-1676.2010.0552.x 20629945

[20] Gentilini F Mancini D Dondi F Ingrá L Turba ME Forni M. Validation of a human immunoturbidimetric assay for measuring canine C-reactive protein. Vet Clin Path. (2005) 34:318.24798319

[21] Christensen M Jacobsen S Ichiyanagi T Kjelgaard-Hansen M. Evaluation of an automated assay based on monoclonal anti-human serum amyloid A (SAA) antibodies for measurement of canine, feline, and equine SAA. Vet J. (2012) 194:332–7. 10.1016/j.tvjl.2012.05.007 22704135

[22] Jansen EHJM Beekhof PK Schenk E. Long term stability of parameters of lipid metabolism in frozen human serum: triglycerides, free fatty acids, total-, HDL- and LDL-cholesterol, apolipoprotein-A1 and B. J Mol Biomark Diagn. (2014) 5:4. 10.4172/2155-9929.1000182

[23] Gislefoss RE Grimsrud TK Mørkrid L. Stability of selected serum hormones and lipids after long-term storage in the Janus Serum bank. Clin Biochem. (2015) 48:364–99. 10.1016/j.clinbiochem.2014.12.006 25523301

[24] Troìa R Giunti M Goggs R. Plasma procalcitonin concentrations predict organ dysfunction and outcome in dogs with sepsis. BMC Vet Res. (2018) 14:1–9. 10.1186/s12917-018-1427-y 29580242 PMC5870177

[25] Golucci APBS Marson FAL Ribeiro AF Nogueira RJN. Lipid profile associated with the systemic inflammatory response syndrome and sepsis in critically ill patients. Nutrition. (2018) 55:7–14. 10.1016/j.nut.2018.04.007 29960160

[26] Bourika V Hantzi E Michos A Margeli A Papassotiriou I Siahanidou T. Clinical value of serum amyloid-A protein, high-density lipoprotein cholesterol and apolipoprotein-A1 in the diagnosis and follow-up neonatal sepsis. Pediatr Infect Dis J. (2020) 39:749–55. 10.1097/INF.0000000000002682 32251257

[27] Milanović Z Vekić J Radonjić V BoŽović AI Zeljković A Janac J. Association of acute Babesia canis infection and serum lipid, lipoprotein, and apoprotein concentrations in dogs. J Vet Intern Med. (2019) 33:1686–94. 10.1111/jvim.15537 31175698 PMC6639482

[28] Hamacher L Dörfelt R Müller M Wess G. Serum cardiac troponin I concentrations in dogs with systemic inflammatory response syndrome. J Vet Intern Med. (2015) 29:164–70. 10.1111/jvim.12474 25619514 PMC4858082

[29] Spapen HD Jacobs R Honoré PM. Sepsis-induced multi-organ dysfunction syndrome-a mechanistic approach. J Emerg Crit Care Med. (2017) 1:27. 10.21037/jeccm.2017.09.04

[30] Kenney EM Rozanski EA Rush JE deLaforcade-Buress AM Berg JR Silverstein DC. Association between outcome and organ system dysfunction in dogs with sepsis: 114 cases (2003–2007). J Am Vet Med Assoc. (2010) 236:83–7. 10.2460/javma.236.1.83 20043806

[31] Zollo AM Ayoob AL Prittie JE Jepson RD Lamb KE Fox PR. Utility of admission lactate concentration, lactate variables, and shock index in outcome assessment in dogs diagnosed with shock. J Vet Emerg Crit Care. (2019) 29:505–13. 10.1111/vec.12868 31290240

[32] Saint-Pierre LM Hopper K Epstein SE. Retrospective evaluation of the prognostic utility of plasma lactate concentration and serial lactate measurements in dogs and cats presented to the emergency room (January 2012–December 2016): 4863 cases. J Vet Emerg Crit Care. (2022) 32:42–9. 10.1111/vec.13106 34343401

[33] Lee SH Park MS Park BH Jung WJ Lee IS Kim SY. Prognostic implications of serum lipid metabolism over time during sepsis. Biomed Res Int. (2015) 2015:789298. 10.1155/2015/789298 26351639 PMC4553311

